# Comparing Transcatheter Tricuspid Valve Intervention With Medical and Surgical Management: A Systematic Review and Meta-Analysis

**DOI:** 10.7759/cureus.83105

**Published:** 2025-04-28

**Authors:** Manav Patel, Muhammad Hamza Nasim, E. Lucano Gómez-Sauceda, Nithin Karnan, Kulsum Farooqi, Tejbir S Monga, Kanika Sood, Nausheen Dhanani, Elmer V Jiménez, Jorge Manuel Aldea Saldaña

**Affiliations:** 1 Internal Medicine, Smt. Nathiba Hargovandas Lakhmichand (NHL) Municipal Medical College, Ahmedabad, IND; 2 Internal Medicine, Lahore Medical and Dental College, Lahore, PAK; 3 Cardiology, Autonomous University of Nuevo León, San Nicolás de los Garza, MEX; 4 Internal Medicine, K.A.P. Viswanatham Government Medical College, Tiruchirappalli, IND; 5 Cardiology, Shadan Institute of Medical Sciences, Hyderabad, IND; 6 Internal Medicine, Spartan Health Sciences University, Vieux Fort, LCA; 7 Cardiology, Guru Gobind Singh Medical College and Hospital, Faridkot, IND; 8 Cardiology, Windsor University School of Medicine - St. Kitts Medical School, Cayon, KNA; 9 Cardiology, Peruvian University of Applied Sciences, Lima, PER

**Keywords:** transcatheter approach, transcatheter heart valve, transcatheter intervention, transcatheter tricuspid valve, transcatheter tricuspid valve intervention, tricuspid regurgitation, tricuspid valve, tricuspid valve repair, tricuspid valve replacement, valvular heart disease

## Abstract

Transcatheter tricuspid valve intervention (TTVI) is rapidly emerging as an attractive option for patients with tricuspid regurgitation (TR). This study aims to compare the outcomes of transcatheter interventions for TR with conventional surgical and medical management strategies. We conducted a systematic review and meta-analysis that included 11 reports from 10 observational studies. One study compared both TTVI versus medical treatment and TTVI versus surgical treatment. The studies were retrieved through a literature search of PubMed, Scopus, and Embase from their inception until April 2024. The review followed the updated Preferred Reporting Items for Systematic reviews and Meta-Analyses (PRISMA) guidelines. Out of six, four studies comparing TTVI with surgical therapy were included in the analysis (due to overlapping populations), while the remaining studies were included in the review. Additionally, five studies comparing TTVI with guideline-directed medical therapy (GDMT) were also incorporated. Patients who underwent TTVI had significantly lower 30-day mortality rates (odds ratio (OR): 0.32, 95% confidence interval (CI): (0.20, 0.50), p < 0.00001), shorter hospital stays (mean difference (MD): -7.33, 95% CI (-8.23, -6.43), p < 0.0001), and lower rates of acute kidney injury (OR: 0.56, 95% CI (0.49, 0.64), p < 0.00001), respiratory complications (OR: 0.45, 95% CI (0.34, 0.60), p = 0.00001), and post-operative cardiogenic shock (OR: 0.31, 95% CI (0.06, 1.53), p = 0.15) when compared to surgical management. TTVI was consistently superior to medical therapy in all included studies, reducing both mortality and heart failure-related hospitalizations. Early intervention with TTVI in patients with low-to-moderate Tricuspid Regurgitation Integrated Score (TRI-SCORE) (recently validated externally, this score captures key outcome drivers, offering a simple and accurate way to predict post-operative mortality and guide the management of patients with TR) was associated with improved outcomes compared to medical therapy alone. Although the available evidence is limited by selection bias and lack of control for confounders, it suggests that TTVI is effective in older, high-risk patients who are considered unsuitable for surgery. Additionally, it shows the superiority of TTVI over medical therapy alone. Future research is necessary to define the optimal candidate profiles for TTVI.

## Introduction and background

Tricuspid regurgitation (TR) is a disease of the heart valve that leads to the backflow of blood into the right atrium; this is due to some factors that lead to the enlargement of the right ventricle. In the United States of America, the prevalence of TR for men and women is 1.5 and 5.6%, respectively, mainly due to functional TR [1]. It is commonly seen in women and older individuals, and the prevalence is increasing, which leads to an increase in mortality [2]. It has also been associated with increased heart failure-related hospitalisations, poor functional status, and quality of life [3]. TR is a common finding in echocardiography that frequently presents in women. It is classified into primary and secondary/functional TR. Primary TR is seen in congenital diseases due to lithium toxicity, and right heart endocarditis is seen in IV drug users [4]. Functional or secondary TR is caused by pulmonary hypertension and left heart failure that leads to structural changes in the right ventricle [4].

Symptomatic TR must be treated, or else it can be fatal. The treatment depends on the stage of the disease, which is determined based on the symptoms and echocardiogram findings. Medical treatment, such as diuretic drugs, is given in the initial stage of the disease, which reduces both central venous and pulmonary congestion and leads to a reduction of symptoms due to volume overload [5]. Surgery was preferred for severely symptomatic patients until recently, and the increased mortality rate for the operative approach gained attention. This led to the development of a safer, less invasive percutaneous approach, primarily transcatheter tricuspid valve intervention (TTVI) [5].

TTVI is considered safe with less post-op morbidity, inpatient mortality, and resources used when compared to surgical repair [6]. It is less invasive and completely bypasses the need for any cardiovascular bypass [7]. On the other hand, surgical tricuspid valvular interventions have high perioperative mortality rates. TTVI is new and needs more clinical trials and studies to develop a better understanding and guideline-based protocol for managing TR patients [8].

Guideline-directed medical therapy (GDMT) and TTVI changed the severity of TR. GDMT is mostly confined to renin-angiotensin system inhibitors, beta-blockers, mineralocorticoid receptor antagonists, and sodium-glucose cotransporter 2 inhibitors, and patients are often seen in clinics or at hospitals for intravenous diuretics [5]. TTVI in high-risk patients with severe TR showed lower rates of re-hospitalisations and death rates when compared to the GDMT group.

In this study, we analyze the best available treatment for TR by comparing TTVI with surgical or medical interventions.

## Review

Materials and methods

Literature Search

We used PubMed, Scopus, and Embase to carry out relevant searches. We formulated the search terms based on the Population, Intervention, Comparator, and Outcome (PICO) framework. We applied the following search strategy to retrieve articles presenting the included terms in their title or abstract: “Tricuspid Regurgitation” OR “TR” OR “Tricuspid regurg” AND “Transcatheter” AND “surgical repair” OR “Tricuspid valve repair” OR “medical management” OR “conservative”. The retrieved studies were screened after reading the abstracts. Duplicates were also omitted using Zotero (Corporation for Digital Scholarship (CDS), Washington, D.C., USA) from the total number of titles and abstracts accumulated. All the studies selected for this analysis were chosen after applying the inclusion and exclusion criteria through the different screening stages. The studies were screened using Rayyan (Qatar Computing Research Institute, Qatar). The results were initially screened through the titles and abstracts by three independent reviewers. All conflicts were meticulously reviewed and resolved by another author. The final selection of studies was determined using the pre-specified inclusion and exclusion criteria. This study was conducted in line with the Updated 2020 Version of the Preferred Reporting Items for Systematic Reviews and Meta-Analyses (PRISMA) guidelines [8].

Inclusion and Exclusion Criteria

Randomized controlled trials, cohort studies, and case-control studies were considered eligible. The inclusion criteria were (1) cohort (in English) studies comparing differences based on various treatment modalities for TR; (2) studies reporting at least one treatment outcome such as all-cause mortality, hospital stay, and perioperative complications; (3) adults >18 years of age; (4) one of the comparison groups is transcatheter treatment; (5) adults with TR corrected with surgical repair or a conservative medical approach as the initial treatment in the control group. The exclusion criteria were (1) systematic reviews, (2) meta-analyses, (3) narrative reviews, (4) editorials, (5) study protocols, (6) case reports, (7) commentaries, (8) single-arm studies, (9) patients under the age of 18, and (10) studies published in languages other than English.

Data Extraction and Outcomes

Four independent investigators performed data extraction. The collected data was then reviewed, and two additional independent reviewers resolved any conflicts to ensure accuracy and consistency. For categorical data, the number of events and total numbers were extracted for each group, while continuous data were recorded as means and standard deviations. If the data were not reported as mean and standard deviation, the method by Wan et al. [9] was used to estimate the mean and standard deviation. The main outcomes of interest included mortality, length of in-hospital stay, blood transfusion, acute kidney injury (AKI), heart failure rehospitalization, quality of life, and improvement in TR symptoms. The extracted data also included the baseline data for the included population.

Quality Assessment

To evaluate the quality of the included studies, we utilised the Newcastle-Ottawa Scale to assess the risk of bias. Two independent reviewers conducted the quality assessment by thoroughly reviewing the articles for adherence to selection criteria, comparability, and outcomes. Regarding follow-up length, we defined it as adequate at one year of follow-up. A follow-up was deemed sufficient if data loss in the patient cohort did not exceed 20%.

Statistical Analysis

This meta-analysis was conducted in line with the guidelines recommended by the Cochrane Collaboration and Meta-analysis of Observational Studies in Epidemiology (MOOSE) [10]. Data analysis was conducted using Review Manager software version 5.4.1 (The Cochrane Collaboration, Oxford, UK). The Mantel-Haenszel random-effects model was utilised to calculate the risk ratio (RR) and corresponding 95% confidence intervals (CIs) for binary outcomes. A p-value of less than 0.05 was considered statistically significant.

Results

Included Studies

**Figure 1:**
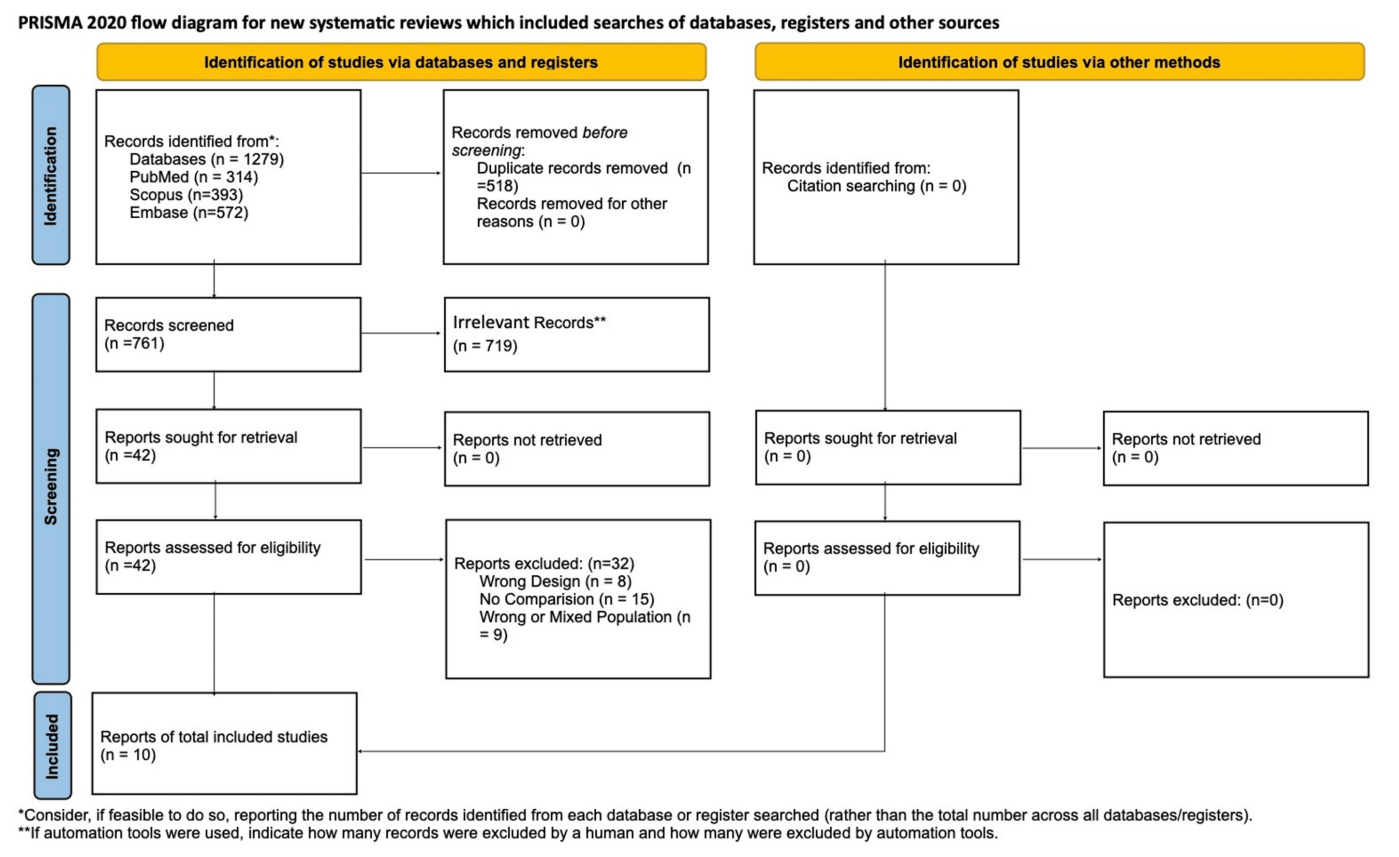
The Preferred Reporting Items for Systematic Reviews and Meta-Analyses (PRISMA) flowchart showing the steps in evaluating retrieved records and selecting studies for inclusion.

In this systematic review and meta-analysis, we included the comparative outcomes of patients undergoing TTVI versus surgical treatment. A total of 10 studies were included: six studies compared TTVI with surgical therapy, five studies compared TTVI with medical therapy, and one study evaluated TTVI against both medical and surgical treatments. The results of the quality assessment are summarized in Table 1. All studies have moderate-to-good quality. Figure 1 shows the PRISMA flowchart highlighting the process of screening. Additionally, five studies investigating the differences between TTVI and conservative medical treatment were included in the systematic review. Tables 2-3 provide a summary of the included studies.

**Table 1:** Risk of bias assessment using the New Castle-Ottawa Scale (follow-up length was determined to be 12 months, adequacy of follow-up meant less than 10% loss at 12 months). ★ indicates that the paper satisfies the conditions; 0 indicates that the paper does not satisfy the conditions.

Study ID	Selection				Comparability		Outcome			Score
	Representativeness of the Exposed (Transcatheter)	Selection of Non-exposed (Med/Surg)	Ascertainment of Exposure	Outcome of Interest Not Present at Start of Study	Main Factor	Additional Factor	Assessment	Follow-Up Length	Adequacy of Follow-Up	
Arnold et al. [3]	★	★	★	★	★	0	★	★	0	7
Cai et al. [5]	★	★	★	★	★	0	★	★	0	7
Mohamed et al. [6]	★	★	★	★	★	★	★	★	0	8
Sorajja et al. [7]	★	★	★	★	★	0	★	★	0	7
Wang et al. [11]	★	★	★	★	★	0	★	★	0	7
Taramasso et al. [12]	★	★	★	★	★	★	★	★	0	8
Dreyfus et al. [13]	★	★	★	★	★	★	★	★	0	8
Lawlor et al. [14]	★	★	★	★	★	0	★	★	0	7
Khalid et al. [15]	★	★	★	★	★	★	★	★	0	8
Ma et al. [16]	★	★	★	★	★	0	★	★	0	7

**Table 2:** Summary of studies comparing TTVI versus surgery for TR. TTVI: transcatheter tricuspid valve intervention; TR: tricuspid regurgitation; OR: odds ratio; TRI-SCORE: Tricuspid Regurgitation Integrated Score

Study ID	Location	Study Duration	No. of Patients		Main Conclusion
			TTVI	Surgical	
Mohamed et al. 2023 [6]	United States	2016-2020	1520	2920	Patients undergoing TTVI had reduced inpatient mortality, fewer clinical complications, and lower resource utilization.
Khalid et al. 2022 [15]	United States	2015-2019	155	715	Surgical treatment of TR is associated with significantly higher mortality, longer length of stay, and higher total charges compared to TTVI.
Ma et al. 2023 [16]	China	2016-2018	14	26	Patients who underwent TTVI experienced lower mortality, fewer periprocedural complications, and reduced resource use compared to surgery.
Lawlor et al. 2022 [14]	Columbia	2011-2021	64	58	Surgery (OR: 8.75, p = 0.0002), compared to TTVI and baseline central venous pressure (CVP) (OR: 1.12, p = 0.016), was strongly associated with in-hospital mortality from cardiogenic shock.
Wang et al. 2023 [11]	United States	2016-2018	84	86	Patients undergoing TTVI had reduced inpatient mortality, fewer clinical complications, and lower resource utilization.
Dreyfus et al. 2023 [13]	Multicenter in Europe	2021-2023	645	551	Patients with a low to moderate TRI-SCORE significantly benefited from early and successful surgical or transcatheter interventions, leading to improved 2-year survival.

**Table 3:** Summary of studies comparing TTVI versus conservative medical treatment for TR. TTVI: transcatheter tricuspid valve intervention; TR: tricuspid regurgitation; TRI-SCORE: Tricuspid Regurgitation Integrated Score; NYHA: New York Heart Association; HF: heart failure;

Study ID	Design	Study Population (TTVI/Medical)	Main Study Endpoint	Study Conclusion
Arnold et al. 2024 [3]	TRILUMINATE Randomized Controlled Trial	TTVI: 175/Medical: 175	Kansas City Cardiomyopathy Questionnaire (KCCQ)	TTVI improved health status at 1 month and 1 year compared to medical therapy. At 1 year, more TTVI patients (74.8% versus 45.9%) were alive and well (p < 0.001). Benefits decreased with higher baseline KCCQ scores and were linked to TR reduction, which correlated with lower 1-year mortality and HF hospitalization.
Cai et al. 2020 [5]	Retrospective Cohort Study	TTVI: 53/Medical: 71	NYHA class, Survival, HF hospitalization, 6-minute walk distance	TTVI led to significant improvements in NYHA functional class and 6-minute walk distance (p < 0.001). In contrast, patients on Medical therapy had lower survival rates (46.9% vs. 75.1%, p = 0.047) and higher HF hospitalization (p = 0.027).
Sorajja et al. 2023 [7]	TRILUMINATE Randomized Controlled Trial	TTVI: 175/Medical: 175	Hierarchical composite of all-cause death, HF hospitalization, and quality of life	TTVI reduced severe tricuspid regurgitation and was associated with improved quality of life at 1 year. The nature of the composite outcome may need caution with interpretation in terms of impact on mortality.
Taramasso et al. 2019 [12]	Matched Prospective Cohort Study	TTVI: 472/Medical: 1179	One-year mortality. HF hospitalization.	TTVI in selected high-risk patients with symptomatic severe tricuspid regurgitation is associated with relatively low mortality and rehospitalization rates at 1 year.
Dreyfus et al. 2024 [13]	Prospective Cohort Study	TTVI: 645/Medical: 1217	Two-year survival	Compared to conservative management, early and successful surgical or transcatheter intervention improved 2-year survival in patients with low TRI-SCOREs and, to a lesser extent, those with intermediate TRI-SCOREs. However, no benefit was observed in patients with high TRI-SCOREs.

Surgical Versus TTVI

Demographics: Four out of six studies [6,13,14,16] involving a total of 5798 patients were included in this meta-analysis, of which 2243 belonged to the transcatheter group and 3555 belonged to the surgical group. The baseline age of the population was significantly higher in the surgical group compared to the transcatheter group (mean difference (MD): 7.45, 95% CI (2.89, 12.01), p = 0.001, I^2 ^= 97%).

**Figure 2:**
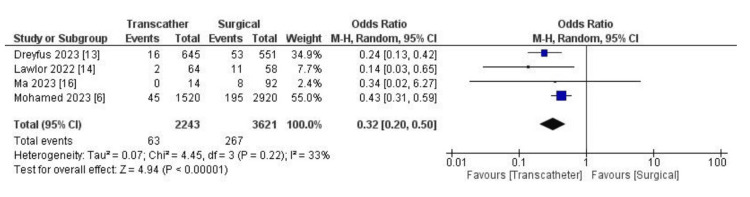
Forest plot showing differences in 30-days mortality between TTVI and surgical management. [6,13,14,16] TTVI: transcatheter tricuspid valve intervention

**Figure 3:**
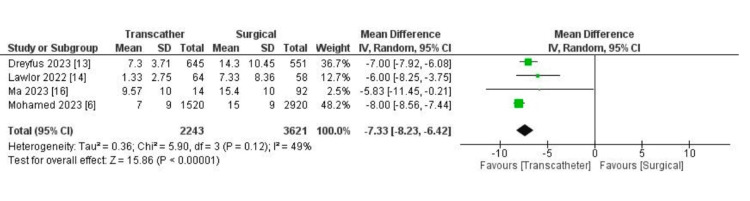
Forest plot showing differences in in-hospital stay between TTVI and surgical management. [6,13,14,16] TTVI: transcatheter tricuspid valve intervention

Endpoint outcomes: The odds of 30-day mortality were considerably lower in the transcatheter group. Analysis revealed statistically significantly fewer adverse outcomes in the transcatheter group (OR: 0.32, 95% CI (0.20, 0.50), p < 0.00001, I^2 ^= 33%) (Figure 2). Regarding the length of the hospital stay, we found a statistically significant shorter length of stay in the transcatheter group (MD: -7.33, 95% CI (-8.23, -6.42), p < 0.0001, I^2 ^= 49%) (Figure 3).

AKI occurred at a significantly lower rate in patients who underwent transcatheter repair compared to the patients who underwent surgical treatment (OR: 0.56, 95% CI (0.49, 0.64), p < 0.00001, I^2 ^= 0%). The incidence of respiratory complications was slightly less frequent in the transcatheter group. The difference was statistically significant, albeit small (OR: 0.45, 95% CI (0.34, 0.60), p = 0.00001, I^2 ^= 0%).

**Figure 4:**
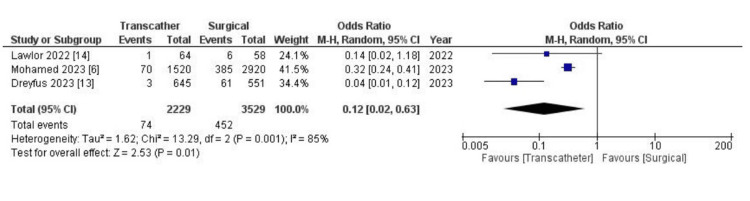
Forest plot showing differences in rates of permanent pacemaker implantation between TTVI and surgical management. [6,13,14] TTVI: transcatheter tricuspid valve intervention

Cardiogenic shock was less frequent in the transcatheter group (OR: 0.31, 95% CI (0.06, 1.53), p = 0.15, I^2 ^= 0%); however, the results were not statistically significant. The incidence of major bleeding post-procedure was non-significant between the TTVI and the surgical group (OR: 0.90, 95% CI (0.61, 1.33), p = 0.60, I^2 ^= 0%). Also, atrio-ventricular block post-procedure was significantly less frequent in the TTVI group (OR: 0.09, 95% CI (0.02, 0.52), p = 0.007, I^2 ^= 0%). Similarly, the TTVI group had significantly lower rates of permanent pacemaker implantation (OR: 0.12, 95% CI (0.02, 0.63), p = 0.01, I^2 ^= 85%) (Figure 4).

Medical Versus TTVI

In terms of survival benefits, TTVI demonstrates significant advantages over medical conservative approaches. Taramasso et al. [12] reported lower one-year mortality rates with TTVI (23% vs. 36%, p = 0.001) and reduced rehospitalization rates (26% vs. 47%, p < 0.0001). Similarly, Cai and colleagues [5] demonstrated that TTVI with GDMT is associated with significantly improved survival rates (46.9% vs. 75.1%, p = 0.047) and lower incidences of heart failure hospitalization (33.2% vs. 62.7%, p = 0.027), gastrointestinal bleeding (15.58 vs. 4.24 per 100 person-years, p = 0.04), and AKI (36.98 vs. 14.12 per 100 person-years, p = 0.001) compared to GDMT alone. The influence of TRI-SCORE on outcomes after TR management was studied by Dreyfus and colleagues [13]. They observed improved 2-year survival rates among low TRI-SCORE patients undergoing TTVI when compared to medical treatment (93% vs. 79%, p = 0.0002), while in high Tricuspid Regurgitation Integrated Score (TRI-SCORE) patients, TTVI did not show a significant survival benefit.

Regarding quality of life and functional capacity improvements, TTVI consistently outperforms medical therapy. Arnold et al. [3] found significant health status improvements with TTVI at one month (mean Kansas City Cardiomyopathy Questionnaire (KCCQ) score difference: 9.4 points, 95% CI: 5.3-13.4) and one year (MD: 10.4 points, 95% CI: 6.3-14.6), with a higher proportion of patients reporting better outcomes (74.8% vs. 45.9%, p < 0.001). The KCCQ is a validated, self-administered questionnaire used to assess health status in individuals with heart failure, with scores ranging from 0 to 100, where higher scores indicate better health. Cai et al. also demonstrated substantial enhancements in New York Heart Association (NYHA) functional class and six-minute walk distance (p < 0.001) with TTVI [5].

The TRILUMINATE randomized trial showed significant reductions in TR severity with TTVI at 30 days (87.0% vs. 4.8%, p < 0.001) compared to medical therapy [7]. The study also highlighted a high safety profile with 98.3% of TTVI patients free from major adverse events at 30 days, emphasising the procedural safety and efficacy of TTVI in clinical practice.

In summary, TTVI emerges as a superior treatment option over medical conservative therapy for patients with severe TR, offering substantial improvements in survival rates, quality of life, functional capacity, and procedural safety. It is crucial to note, nonetheless, that the studies included in this meta-analysis focused mostly on the short-term results; therefore, in order to prove that TTVI is safer and more effective than surgical or medicinal care, long-term results must be examined. Furthermore, additional data is required to evaluate the long-term results for patients receiving TTVI. These findings underscore the clinical benefits of TTVI, however, primarily through observational evidence. Tables 2-3 summarise the conclusions of the reviewed studies.

Discussions

Transcatheter valve interventions have undergone significant advances and are continuously evolving. These advances include the development of various transcatheter devices, improved designs of artificial valves, and tailored access routes, which make them applicable in elective, urgent, and emergent settings [17-19]. The use of transcatheter interventions in tricuspid disease is still developing due to the lower prevalence of TR compared to other valve diseases. This systematic review and meta-analysis aim to summarise the evidence on how TTVI compares to surgical and medical management of TR.

This meta-analysis of outcomes in patients with TR who received either a transcatheter or surgical intervention shows that the TTVI group has lower odds of 30-day mortality and a shorter length of hospital stay. Additionally, the TTVI group has a significantly lower complication rate. The main drivers of adverse outcomes include the timing of clinical presentation, TR severity, and its effects on the right ventricle, kidneys, and liver [8]. Pre-procedural hemodynamics, age, and other comorbidities also play an essential role in determining whether TTVI or surgical interventions are more appropriate. In line with previous studies [11], our study also found that patients selected for TTVI had higher comorbidities, including heart failure (78.88% in the TTVI group vs. 76.44% in the surgical group), hyperlipidemia (54.8% vs. 48.5%), renal failure (62.6% vs. 21.5%), and chronic obstructive pulmonary disease (19.72% vs. 16.57%). These patients were also significantly older (78.7 (SD = 10.6) vs. 66.5 (SD = 12.2)) than those undergoing surgical interventions.

Our meta-analysis highlights the benefits of TTVI for older, more comorbid patients, as it is associated with significant improvements in mortality rates (Table 2) and reduced occurrences of postoperative complications when compared to surgical interventions [20]. Given the high risk associated with tricuspid surgery [17] and the poor outcomes of conservative therapy [5], TTVI has emerged as a viable, less invasive alternative associated with better outcomes. However, a major question remains: the optimal timing for initiating TTVI.

Although the feasibility and initial efficacy of TTVI have been well-documented, data on clinical outcomes with extended follow-up are still scarce. Currently, transcatheter leaflet repair is the most commonly used method for treating certain patients with TR. The procedure offers good safety and a reported procedural success rate of 72-86% (TR grade ≤2+) [21]. Most TTVI techniques are still in the early stages of research, and there is currently no evidence to support safety and effectiveness comparisons between TTVI and surgical replacement or repair from randomised controlled trials or other high-caliber clinical studies. Orban et al. [22] demonstrated the impact of TTVI, showing a 22% reduction in the average yearly hospitalisation rate for heart failure patients, from 1.21 to 0.95 per patient per year (p = 0.02). Additionally, there was a sustained decrease in TR grade (72% of patients had moderate or lower TR) and a significant improvement in NYHA class (grades I-II in 67% of patients at one year compared to 9% at baseline; p < 0.001). The progressive nature of TR and its long-term impact on cardiac function are critical factors that are sometimes overlooked. The primary determinant of postoperative outcomes is the clinical presentation [23-25]. The 10% in-hospital mortality rate following isolated tricuspid valve surgery is partly attributable to delayed management following right ventricular remodeling or renal and hepatic failure.

The recently developed TRI-SCORE, which is based on eight clinical, biological, and echocardiographic parameters (age, NYHA functional class, right-sided heart failure signs, daily dose of furosemide, glomerular filtration rate, total bilirubin level, left ventricular ejection fraction, and right ventricular function), is useful in predicting in-hospital mortality following isolated tricuspid valve surgery [13]. The predictive value of the TRI-SCORE has been externally validated [26,27] and has been shown to extend to patients referred for TTVI [28].

The TRI-SCORE can be used to compare outcomes based on the timing of intervention - whether surgical, conservative, or transcatheter. Dreyfus et al. [13] not only highlight poorer postoperative mortality rates for patients with more severe TR but also demonstrate that early intervention, whether surgical or transcatheter, is beneficial.

With the help of new mobile applications to measure the TRI-SCORE and its increased awareness, it has become increasingly important to consider the TRI-SCORE when selecting an intervention for TR patients. Dreyfus et al. [13] support this assumption, showing significant benefits for patients with low- or intermediate-level TRI-SCOREs who underwent transcatheter interventions. However, this survival benefit was not observed in patients with a high TRI-SCORE who underwent transcatheter intervention compared to those who underwent surgery.

One more crucial aspect to consider is the Obesity Paradox, a counterintuitive relationship where a higher body mass index (BMI) can positively impact clinical outcomes. This phenomenon may be due to several mechanisms, such as an increase in total blood volume and cardiac output in individuals with obesity. According to Seo et al. [29], analyses of mortality outcomes in overweight and obese patients undergoing transcatheter aortic valve replacement (TAVR) show a generally favorable trend. These findings may also be applicable to patients undergoing TTVI, potentially leading to improved operative outcomes based on patients' BMI trends. However, there is currently no evidence for this assumption, and further studies are required before assuming whether BMI plays a role in the outcomes for patients undergoing TTVI.

The significance of carefully risk-stratifying TR patients and adopting early intervention is emphasised by these findings. On the other hand, survival in patients with a high TRI-SCORE was similar across surgical, transcatheter, and conservative treatment groups, suggesting no survival benefit at an advanced stage of the illness. In addition to comparing TTVI with surgery, it is essential to consider the advantages TTVI provides over conservative therapy based on TR guidelines. Patients who undergo TTVI rather than medical therapy are typically selected based on a large coaptation gap (≥10 mm), anatomical instability, and poor echocardiographic visualisation of the tricuspid valve [5]. According to Taramasso et al. [12], patients who received TTVI rather than medical therapy had significantly lower one-year mortality (23.3% vs. 36.3%) and a lower rate of rehospitalisation (26.3% vs. 47.3%). A study also found that TTVI had a lower rate of AKI or gastrointestinal bleeding complications compared to medical therapy alone [5]. These findings suggest that TTVI offers better outcomes than medical conservative therapy alone in treating TR.

Based on our systematic review, TTVI appears to offer better short-term outcomes compared to surgical or medical treatments. However, it is crucial to acknowledge the differences in the baseline characteristics of patients selected for surgical therapy versus those who remain on conservative treatment. Additionally, studies included in this meta-analysis focus on short-term outcomes, and it is imperative to assess long-term outcomes to establish the superiority of TTVI over surgical or medical management in terms of safety and effectiveness. More evidence is needed to evaluate the long-term outcomes for patients undergoing TTVI.

Strengths and Limitations

We present a large summary of real-world patient data who underwent a relatively uncommon procedure. The observational nature of studies, though limited by confounders, provides a reflection of real-life scenarios, which is equally important. Furthermore, this study’s strength lies in its focus on a critical clinical issue, offering a comprehensive overview of current evidence on treatment strategies for TR in cardiac patients. The meta-analysis directly compares TTVI with surgical management and evaluates the efficacy of TTVI versus medical therapy. It also highlights key gaps in research, particularly regarding optimal timing for TTVI and the potential role of the TRI-SCORE.

However, several limitations should be noted. The analysis includes only four observational studies comparing TTVI with surgery and five comparing TTVI with medical treatment. The observational design introduces selection bias, limiting the generalizability of results, and the small number of studies underscores the need for more robust evidence. Additionally, the included studies likely involved different types of transcatheter devices (e.g., edge-to-edge repair, annuloplasty, valve replacement), each with distinct success rates and risk profiles. Most studies also reported only short- to mid-term outcomes (e.g., 30-day or 1-year), which may not reflect long-term durability, complications, or survival benefits of TTVI. Additionally, our study mainly focuses on research conducted in the United States, therefore, variations in socio-economic status must be considered when applying findings to patient populations with varying socio-economic backgrounds [30].

Future Recommendations

Future research should involve more standardized patient selection criteria and well-structured protocols. Including comprehensive data, particularly with subgroup analyses regarding the stage of the disease and the influence of different treatment strategies on patient outcomes, is crucial. The optimal timing for TTVI should be further explored through validation studies to enhance decision-making.

Additionally, as transcatheter treatments evolve, there is a shift towards the use of local rather than general anesthesia, alongside shorter hospital stays and reduced 30-day mortality rates [31]. Future studies would benefit from assessing the safety and effectiveness of local anesthesia in the context of TTVI, as this could have significant implications for patient outcomes and procedural costs.

## Conclusions

In conclusion, our results show that patients with TR who underwent transcatheter intervention had lower odds of 30-day mortality, shorter in-hospital stays, and a significantly lower complication rate compared to the surgical group. The existing body of evidence strongly supports the broad applicability and versatility of TTVI in treating a diverse range of patients with TR. However, factors such as the timing and severity of the clinical presentation, TR grade, and its effects on the right ventricle, kidneys, and liver also play a critical role in patient prognosis. These considerations must be carefully evaluated when determining the optimal timing for the procedure, selecting appropriate candidates, and acknowledging the current lack of comprehensive long-term outcome data on TTVI.

While these findings are significant, future studies should aim to optimize the timing of the intervention and further refine procedural protocols to maximize patient outcomes. Healthcare professionals must also carefully consider various factors before opting for TTVI over traditional surgical interventions, ensuring that it remains the most appropriate treatment for each patient. Addressing these issues will help optimize the use of TTVI and solidify its position as a preferred treatment modality in suitable cases.
